# Supplementary material for: Patterns of Intron Gain and Loss in Fungi
Source: PLoS Biol. 2004 Nov 30;2(12):e422. doi: 10.1371/journal.pbio.0020422 (PMC532390; doi:10.1371/journal.pbio.0020422)
Supplement: Table S1 — Also available at http://genes.mit.edu/NielsenEtAl/. (4.3 MB ZIP). [file pbio.0020422.st001.zip › NielsenEtAl/html/1423.html]

AN5123.1.NCU01704.1.MG08569.1.FG00320.1


```
 CLUSTAL W (1.82) Multiple Sequence Alignments - Introns Inserted


Sequence 1: NCU01704.1	392 aa
Sequence 2: FG00320.1	391 aa
Sequence 3: MG08569.1	394 aa
Sequence 4: AN5123.1	388 aa
Alignment Length: 409 aa
Number Identitical Residues: 176 aa
Alignment Score (without introns) 8968


MG08569.1 	MTS-SPFTVRWGIMA~TGGIAET1FAKDLLTDPAI-RDVSDVRHDVVAAASSTSADRATA
NCU01704.1	MASNTPFTLKWGIMA~TGWIAET1FTQDLLTNPATTRDVHDVRHEVVAAASSSSKQRAEE
FG00320.1 	MA---PYIAKWGILA~TGGIAEC1FTKDLLTDPAT-REVDDVQHEIVAVASSSSVDRARD
AN5123.1  	MA---PYNIRWGILA1TGGIADA1FVRDLLLDPST-RSASDISHTVVAVSSSSSASRAEK
          	*:   *:  :***:* ** **:  *.:*** :*:  *.. *: * :**.:**:* .**  

MG08569.1 	FLKKIDAPNKDAKAYGSYAELVADPNVDIVYVATPHSHHFQNAMLALEAGKHVLCEKALT
NCU01704.1	FLVKVKAPS-SAKAYGSYHELVADPEIDIIYVATPHSHHFQNAMLALEAGKNVLCEKALT
FG00320.1 	FIKKIDGPS-SAAVYGSYAELVADPNVNIIYVATPHSHHFQNAMLALEAGKNVLCEKALT
AN5123.1  	FIANTGIPS-PCTAYGSYEALVADPNVDVVYVATPHSHHFQNVMLCLEAGKHVLCEKAFT
          	*: :   *.  . .****  *****:::::************.**.*****:******:*

MG08569.1 	VTAAQTRKMIETAKAKKLFLMEAVWTRYFPLSIKIRDLIKSGAIGSVHRVMA~DFSFNNS
NCU01704.1	VTASQARKLVDMAKKKKVFFMEAVWTRYQPISIKVRELIQSGVIGPVYRVVA1DLSFGND
FG00320.1 	VTVAQTKKLYEVAKSKNLFFMEAVWTRYFPLSIKVRELIQSGVIGNVYRTFA1DLSFNKN
AN5123.1  	VNAQQTRILVETARKKNLFLMEAVWTRYFPLSIEIRELIKNGAIGEVLRVTA~DNSFGGT
          	*.. *:: : : *: *::*:******** *:**::*:**:.*.** * *. * * **.  

MG08569.1 	AADGGLDFDNGNRMVNPDLAGGALLDL~GIYSLTWVFQTLYHTQAAGNKEKPT-VTANVM
NCU01704.1	QPDGTVSFADTHRMVNPDLAGGALLDL~GIYSLTWVFQTMYHLQ--KEKEKPK-VVAAVQ
FG00320.1 	TNSDDLEFPDSNRMVNPDLAGGALLDL~GIYSLTWVFQSLYHVQPEADKEAPN-VVASIN
AN5123.1  	PEE---KFSTEHRMVNKDLAGGALLDL1GIYSLTWVFQTLYHTLPREARKPPSRVSAHIT
          	  .   .*   :**** ********** **********::**  .   :: *. * * : 

MG08569.1 	RYDATGTDETTTVLVGFPEHK---------AVGVAMTSLRVASDVDGKWTAGPTIRIQGS
NCU01704.1	KYH-TGADEATSIIVQWPQHK---------SQGIALTSLRLATGVDKENTGGPGIRIQGS
FG00320.1 	KYR-TGADESTSIICQFPKHR---------SVGIATTSLRIATDVDGHYTGGPAIRIQGS
AN5123.1  	PYHLTGADEATTILLSFPTTTPSNSEHPGESQAVASTSIRVATDPDETNTARPAIRIQGT
          	 *  **:**:*:::  :*   .:.:. ...: .:* **:*:*:. *   *. * *****:

MG08569.1 	GGEIAVYGVAAKPFKFHVL--RKGQQ-EPEVVECPFPKDDKRGGWGHGMYWEADEAARCL
NCU01704.1	GGEIHVTGPAFRPLQYKVI--RGDAGGKIETVDFSIPKDPERD-WGHGMFWEADECARCI
FG00320.1 	KGEIQVTGPAYRPTEYKVI--KSDANGNVEVVTCPIPTDPKRNNWGHGMFWEADECARCL
AN5123.1  	KGEIQVDGPAFRPEVYRIIPVKGQGMGEFKEKKFDFPAN------GRGMYWEADEVARCL
          	 *** * * * :*  ::::. :    .: :     :* :      *:**:***** ***:

MG08569.1 	RDGKLESEGLPHEESIAIMEVMEETLRQGGVKYPELITSDVYDPKSPLNTGKA
NCU01704.1	RDGKKESESLPWEESLVMMEVMEEALKQGGVTYPDLITTDVFDAQSPLNTGKR
FG00320.1 	RDGKKESASMPWSESIAIMEVMDSALKQGGVTYPELITTDVFDANSPLNTGRK
AN5123.1  	RDGKLESETMSLEESIVIMDVMDEARRQNGLTYPEKIESTVYPTQL-------
          	**** **  :. .**:.:*:**:.: :*.*:.**: * : *: .:
```
